# Pharmacological Effects of Panduratin A on Renal Cyst Development in In Vitro and In Vivo Models of Polycystic Kidney Disease

**DOI:** 10.3390/ijms23084328

**Published:** 2022-04-14

**Authors:** Kanlayanee Tonum, Nipitpon Srimai, Napason Chabang, Somsak Fongsupa, Patoomratana Tuchinda, Jacob A. Torres, Thomas Weimbs, Sunhapas Soodvilai

**Affiliations:** 1 Research Center of Transport Protein for Medical Innovation, Department of Physiology, Faculty of Science, Mahidol University, Ratchathewi, Bangkok 10400, Thailand; kanlayanee.ton@mahidol.ac.th (K.T.); nipitpon.sri@student.mahidol.ac.th (N.S.); 2 School of Bioinnovation and Bio-Based Product Intelligence, Faculty of Science, Mahidol University, Ratchathewi, Bangkok 10400, Thailand; napason.cha@mahidol.ac.th; 3 Department of Medical Technology, Faculty of Allied Health Science, Thammasat University Rangsit Campus, Pathumthani 12121, Thailand; fongsupa.mu@gmail.com; 4 Excellent Center for Drug Discovery, Mahidol University, Ratchathewi, Bangkok 10400, Thailand; scptcster@gmail.com; 5 Molecular, Cellular, and Developmental Biology, and Neuroscience Research Institute, University of California, Santa Barbara, CA 93106-9625, USA; jacobtorres@ucsb.edu (J.A.T.); weimbs@ucsb.edu (T.W.)

**Keywords:** ADPKD, cystogenesis, AMP-activated protein kinase (AMPK), cell proliferation, cystic fibrosis transmembrane conductance regulator (CFTR), renal fluid secretion

## Abstract

Renal cyst expansion in polycystic kidney disease (PKD) involves abnormalities in both cyst-lining-cell proliferation and fluid accumulation. Suppression of these processes may retard the progression of PKD. Evidence suggests that the activation of 5′ AMP-activated protein kinase (AMPK) inhibits cystic fibrosis transmembrane conductance regulator (CFTR)–mediated chloride secretion, leading to reduced progression of PKD. Here we investigated the pharmacological effects of panduratin A, a bioactive compound known as an AMPK activator, on CFTR-mediated chloride secretion and renal cyst development using in vitro and animal models of PKD. We demonstrated that AMPK was activated in immortalized normal renal cells and autosomal dominant polycystic kidney disease (ADPKD) cells following treatment with panduratin A. Treatment with panduratin A reduced the number of renal cyst colonies corresponding with a decrease in cell proliferation and phosphorylated p70/S6K, a downstream target of mTOR signaling. Additionally, panduratin A slowed cyst expansion via inhibition of the protein expression and transport function of CFTR. In heterozygous Han:Sprague–Dawley (Cy/+) rats, an animal model of PKD, intraperitoneal administration of panduratin A (25 mg/kg BW) for 5 weeks significantly decreased the kidney weight per body weight ratios and the cystic index. Panduratin A also reduced collagen deposition in renal tissue. Intraperitoneal administration of panduratin A caused abdominal bleeding and reduced body weight. However, 25 mg/kg BW of panduratin A via oral administration in the PCK rats, another non-orthologous PKD model, showed a significant decrease in the cystic index without severe adverse effects, indicating that the route of administration is critical in preventing adverse effects while still slowing disease progression. These findings reveal that panduratin A might hold therapeutic properties for the treatment of PKD.

## 1. Introduction

Polycystic kidney disease (PKD) is a common genetic disease characterized by the development and enlargement of kidney cysts that eventually lead to chronic renal failure [1,2]. Autosomal dominant polycystic kidney disease (ADPKD) is the most common form of PKD. ADPKD is caused by mutations in one of two genes, *PKD1* or *PKD2*, encoding the proteins polycystin-1 (PC1) and polycystin-2 (PC2), respectively. Mutations in PC1 or PC2 result in decreased intracellular Ca^2+^ and a subsequent increase in cAMP [3]. The accumulation of intracellular cAMP activates cyst epithelial-cell proliferation and fluid secretion into the cyst lumen [4,5]. The excessive proliferation of cyst epithelial cells is facilitated by dysregulation of numerous signaling pathways, including the Wnt, B-Raf/MEK/ERK, and mammalian target of rapamycin (mTOR) signaling pathways [6]. Additionally, several lines of evidence illustrate that the cystic fibrosis transmembrane conductance regulator (CFTR) chloride channel is important for regulating fluid accumulation and electrolytes in ADPKD cyst lumens [7]. Small-molecule CFTR inhibitors, such as thiazolidinone inhibitor CFTR-172 and phenyl-derivatized glycine hydrazide CFTR inhibitors, were demonstrated to attenuate cyst expansion in both in vitro and in vivo models of PKD. Moreover, CFTR inhibitors reduced cyst progression and preserved renal function in *Pkd1* knockout mice [8]. Previous studies reveal that nuclear receptors, including peroxisome proliferator–activated receptor gamma (PPARγ) and liver X receptor (LXR), inhibit CFTR-mediated chloride secretion and lead to a decrease in renal cyst progression [9,10,11,12]. This evidence supports the inhibition of CFTR as a potential target for the treatment of PKD.

In PKD animal models, agents which decrease cell proliferation were shown to retard cyst progression [13,14,15,16]. Inhibition of the activity or function of numerous targets, including vasopressin receptors, calcium-sensing receptors, CFTRs, the cell cycle, and mTOR have been proposed for ADPKD treatment [8,13,14,15,16,17,18,19]. Importantly, 5′ AMP-activated protein kinase (AMPK) activation by metformin, an anti-diabetic drug, was found to reduce renal cyst formation and growth through the inhibition of cell proliferation and CFTR-mediated fluid secretion [20]. Moreover, 2-deoxy-D-glucose (2DG), a glucose analog, restored the activation state of AMPK and slowed PKD progression in a PKD mice model [21]. Thus, AMPK activators might be potential therapeutic agents for drug development in PKD treatment.

Panduratin A, a bioactive compound found in *Boesenbergia rotunda*, has been reported to be an activator of AMPK. Panduratin A reduced high fat diet (HFD)–induced obesity via the stimulation of liver kinase B1 (LKB1)–dependent AMPK signaling [22]. In addition, panduratin A was shown to have anti-proliferative and anti-inflammatory properties and additionally regulates apoptosis [23,24]. Since CFTR plays a role in cyst enlargement, it is imperative to investigate whether panduratin A could inhibit CFTR-mediated chloride secretion leading to decreased renal cyst enlargement in PKD models. The present study revealed the pharmacological effects of panduratin A and their corresponding mechanisms on CFTR-mediated chloride secretion and renal cyst enlargement using Madin–Darby canine kidney (MDCK) cell–derived cysts. In addition, the therapeutic potential of panduratin A was investigated in animal models of PKD.

## 2. Results

### 2.1. Panduratin A Treatment Slows in Vitro Cystogenesis

**Figure 1:**
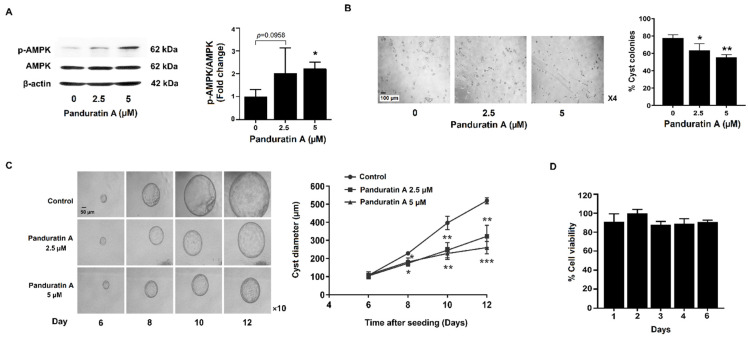
Effect of panduratin A on cystogenesis in MDCK cell–derived cyst model. (**A**) Activation of AMPK in MDCK cells following treatment with panduratin A for 24 h (*n* = 5). (**B**,**C**) MDCK cell–derived cyst colonies (**B**) and cyst growth (**C**) in response to panduratin A. (**C**) Representative micrographs show the same MDCK cysts after exposure to vehicle or 2.5 and 5 µM panduratin A (>20 cysts analyzed/time point). (**D**) Cell viability following treatment with 5 µM panduratin A. Data of (**B**–**D**) are shown as the mean ± SD of 3 independent experiments. * *p* < 0.05, ** *p* < 0.01, and *** *p* < 0.001 compared to control.

First, we tested the effects of panduratin A on AMPK activation in MDCK cells. MDCK cells were incubated with either vehicle or panduratin A (2.5 and 5 µM) for 24 h. Treated MDCK cells were then probed via Western blot to measure levels of phosphorylated AMPK. Panduratin A (5 µM) significantly increased phosphorylated AMPK compared to vehicle, indicating that panduratin A can stimulate AMPK in MDCK cells (Figure 1A). Next, we determined the effect of panduratin A on cystogenesis. As shown in Figure 1B,C, panduratin A significantly reduced the percentage of cyst colonies relative to vehicle. In addition, the mean cyst diameter of the panduratin A–treated group was significantly less than that of the vehicle control. Cell viability was then tested to examine whether panduratin A caused cytotoxicity in MDCK cells. Exposure of MDCK cells to 5 µM panduratin A for 6 days did not affect cell viability (Figure 1D).

### 2.2. Panduratin A Treatment Reduces Cell Proliferation via Inhibition of mTOR Signaling in MDCK Cells

**Figure 2:**
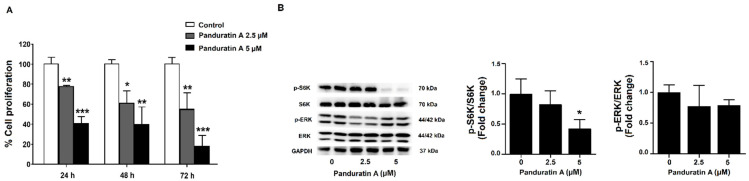
Effect of panduratin A on cell proliferation and intracellular protein signaling of cell proliferation markers in MDCK cells. (**A**) MDCK cells were treated with vehicle or 2.5 or 5 µM panduratin A for 24, 48, and 72 h (*n* = 3). (**B**) Representative and quantitative analysis of S6K and ERK expression in MDCK cells following 24 h treatment (*n* = 5). Data are expressed as the mean of percentage control ± SD. * *p* < 0.05, ** *p* < 0.01, and *** *p* < 0.001 compared to control.

Since panduratin A reduced the number of cyst colonies, we tested whether it affected cell proliferation. Thus, we investigated the effects of panduratin A on the proliferation of MDCK cells using the BrdU cell proliferation assay. As shown in Figure 2A, incubation with panduratin A (2.5 and 5 µM) for 24, 48, and 72 h significantly reduced MDCK cell proliferation compared to vehicle-treated cells. Next, we determined whether the inhibitory effect of panduratin A was mediated by inhibition of the MEK/ERK and mTOR pathways. As shown in Figure 2B, panduratin A slightly reduced phosphorylated ERK. In addition, panduratin A significantly decreased levels of phosphorylated S6K, a downstream target of mTOR.

### 2.3. Panduratin A Inhibits CFTR-Mediated Chloride Secretion in MDCK Cells

**Figure 3:**
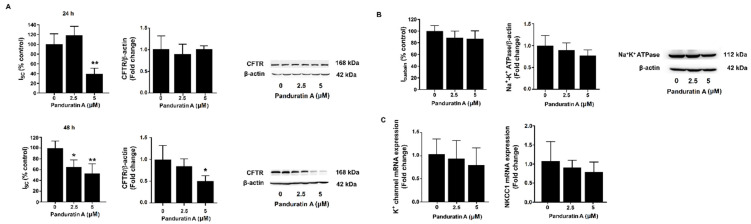
Effect of panduratin A on function and expression of ion transporters. (**A**) CFTR-mediated Cl^-^ secretion (*n* = 10) and CFTR protein expression (*n* = 4–5). (**B**) Na^+^–K^+^ ATPase activity (*n* = 3) and protein expression (*n* = 5), and (**C**) K^+^ channel and NKCC1 mRNA expression (*n* = 3). MDCK cell monolayers were incubated with panduratin A at indicated concentrations for 24 h or 48 h. Data are presented as mean of percentage control ± SD; * *p* < 0.05, ** *p* < 0.01 compared to control.

Considering that panduratin A treatment reduced MDCK-derived cyst growth, we next sought to examine whether the inhibitory effect of panduratin A on cyst growth was a result of CFTR-mediated chloride secretion inhibition. The MDCK cell monolayers grown on Snapwell Inserts were incubated with either vehicle or panduratin A (2.5 or 5 µM) for 24 and 48 h, followed by measurement of the short-circuit current (Isc). The results showed that treatment of the cell monolayers with 2.5 µM panduratin A for 48 h, but not for 24 h, significantly decreased the Isc. However, exposure the cell monolayers to 5 µM panduratin A significantly decreased Isc at both 24 h and 48 h. Interestingly, Western blot analysis revealed that panduratin A for 24 h did not alter CFTR protein expression. However, increase in exposure time of panduratin A caused a significant reduction in CFTR protein expression (Figure 3A). Next, we tested the involvement of Na^+^–K^+^ ATPase in the inhibitory effect of panduratin A on the Isc. As shown in Figure 3B, incubation of MDCK cell monolayers with panduratin A (2.5 and 5 µM) did not affect protein expression or the transport function of Na^+^–K^+^ ATPase, which was represented by ouabain-sensitive current (Iouabain). In addition, panduratin A did not significantly alter K^+^ channel and NKCC1 mRNA expression (Figure 3C).

### 2.4. Panduratin A Treatment Reduces the Proliferation of Human ADPKD Cells

**Figure 4:**
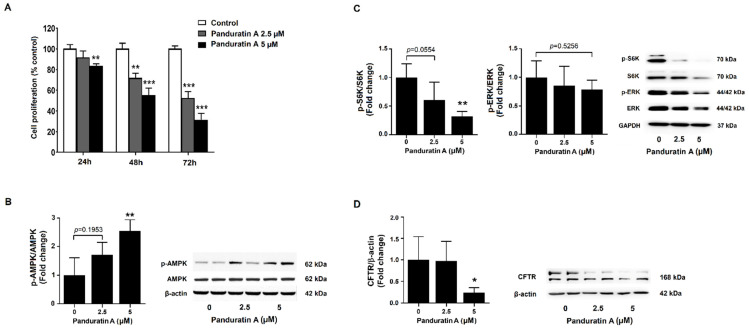
Effect of panduratin A on cell proliferation in ADPKD cells. (**A**) Cell proliferation was examined after 24, 48, and 72 h of incubation (*n* = 3). (**B**–**D**) Representative immunoblots and quantitative protein expressions following treatment of cells with panduratin A at indicated concentrations for 24 h (*n* = 5). Data are shown as mean ± SD. * *p* < 0.05, ** *p* < 0.01, and *** *p* < 0.001 compared to control.

We determined the effect of panduratin A on the proliferation of WT9-12 cells, which were derived from cystic tubules of human ADPKD [25]. WT9-12 cells were incubated with vehicle or panduratin A (2.5 and 5 µM) for 24–72 h and examined for differences in cell proliferation. The results showed that panduratin A significantly reduced proliferation of ADPKD cells compared to the vehicle-treated group (Figure 4A). Treating ADPKD cells with 5 µM panduratin A for 24 h significantly increased levels of phosphorylated AMPK (Figure 4B). Panduratin A significantly decreased levels of phosphorylated S6K, but not ERK, compared to vehicle-treated cells (Figure 4C). Moreover, panduratin A significantly reduced protein expression of CFTR compared to that of vehicle-treated cells (Figure 4D).

### 2.5. Effect of Panduratin A on Renal Cyst Progression in PKD Rat Models

**Figure 5:**
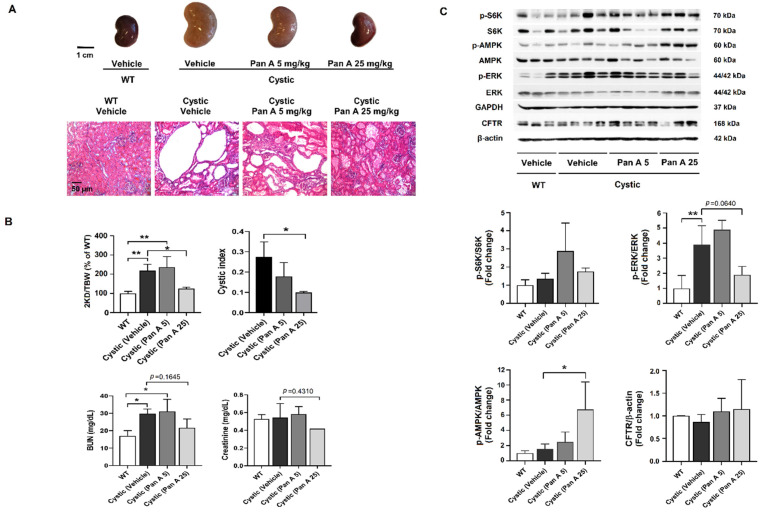
Effect of panduratin A on renal cyst progression in PKD rats. (**A**) Representative images of kidney and H&E staining of histological kidney sections from wild-type (WT), vehicle-treated cystic (Han:SPRD) rats, and panduratin A ((Pan A), 5 mg/kg BW or 25 mg/kg BW)–treated cystic rats. (**B**) Percentage kidney weight to total body weight (2KD/TBW), cystic index, and parameter assessment of renal function in cystic rats. (**C**) Representative immunoblots and band intensities of AMPK, mTOR/S6K, ERK, and CFTR. Data are shown as mean ± SD, *n* = 3–4 rats per group, * *p* < 0.05 and ** *p* < 0.01.

To confirm that panduratin A has an inhibitory effect on renal cyst progression in ADPKD, male Han:SPRD rats with PKD (Cy/+) were given daily intraperitoneal (i.p.) injections of vehicle or panduratin A (5 or 25 mg/kg BW) for 5 weeks. The doses of panduratin A were selected based on our previous study [26]. As shown in Figure 5A,B, administration of panduratin A at the higher dose of 25 mg/kg BW was able to decrease kidney weight and cystic area relative to vehicle-treated cystic rats. Additionally, panduratin A (25 mg/kg BW) showed a tendency toward reduction of blood urea nitrogen (BUN) and serum creatinine (Cr) levels compared to vehicle-treated cystic rats (Figure 5B). Furthermore, we next examined the effects of panduratin A on the expression of proteins involved in cyst progression. As shown in Figure 5C, rats treated with panduratin A (25 mg/kg BW) tended to exhibit a reduction in phosphorylated ERK. Alternatively, panduratin A treatment did not alter phosphorylated S6K and CFTR. As hypothesized, in rats treated with panduratin A, kidney tissue exhibited higher AMPK phosphorylation levels than the kidneys of vehicle-treated rats. Although the higher dose of panduratin A treatment showed efficacy on cyst progression in (Cy/+) Han:SPRD rats, it provided unexpected adverse effects such as darkened testis, abdominal bleeding, and a decrease in body weight (Supplement Table S1).

**Figure 6:**
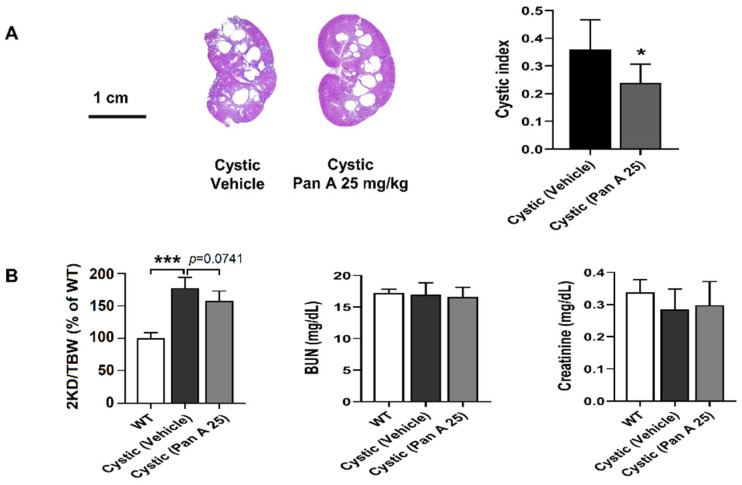
Effect of panduratin A on renal cyst progression in ARPKD rats. (**A**) H&E staining of histological kidney sections and cystic index from PCK rats treated with vehicle and panduratin A (Pan A) 25 mg/kg BW. (**B**) Percentage kidney weight to total body weight (2KD/TBW) and parameter assessment of renal function in wild-type (WT) and cystic (PCK) rats. Data are shown as mean ± SD, *n* = 4 and 6 per group for WT and PCK rats, respectively, * *p* < 0.05 and *** *p* < 0.001.

We additionally evaluated the effect of panduratin A on PCK rats, an orthologous rat model of ARPKD that shows the phenotype of ADPKD [27]. The effect on kidney weight, renal cystic area, and renal function parameters, including BUN and serum Cr, were investigated following treatment by oral gavage with panduratin A at 25 mg/kg BW starting at 4 weeks of age. As shown in Figure 6, treatment with 25 mg/kg of panduratin A for 8 weeks showed a significantly decrease in renal cystic index and showed a trend of reduced percentage of kidney weight to body weight compared to vehicle-treated rats. Although the cysts were found in kidneys of PKC rats, we did not detect a reduction of kidney function, as shown by normal BUN and serum Cr levels. In addition, PCK rats treated with panduratin A did not exhibit altered BUN and serum Cr levels, even though the higher dose of panduratin A treatment showed adverse effects in (Cy/+) Han:SPRD rats. However, these adverse effects were not found in PCK rats following oral administration of panduratin A at the same dose.

### 2.6. Effect of Panduratin A on Interstitial Fibrosis in Kidneys PKD Rats

**Figure 7:**
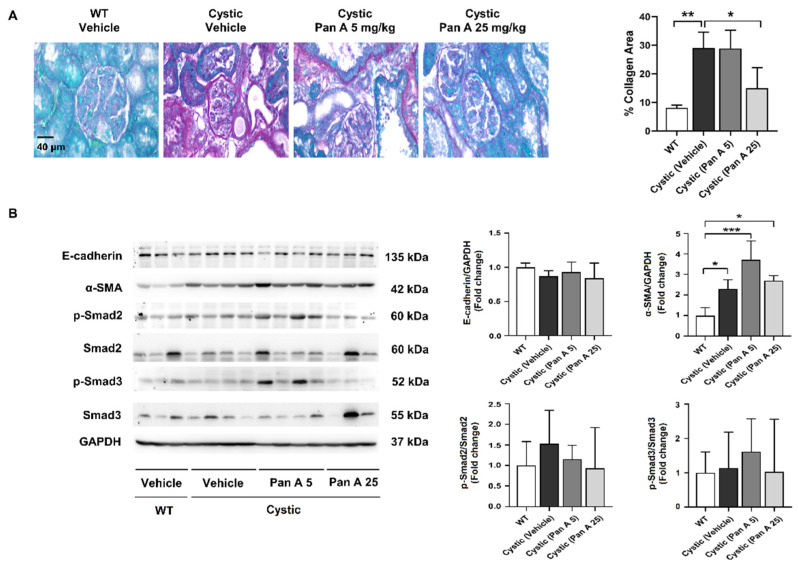
Effect of panduratin A on renal collagen deposition and fibrosis-related proteins in PKD rats. (**A**) Representative images of Sirius Red–stained kidney sections from wild-type (WT) and cystic (Han:SPRD) rats treated with vehicle or panduratin A (PanA) and percentage collagen area. (**B**) Representative and quantitative immunoblots for fibrosis-related proteins. Data are shown as mean ± SD, *n* = 3–4 rats per group, * *p* < 0.05, ** *p* < 0.01, and *** *p* < 0.001.

PKD pathogenesis also typically includes interstitial fibrosis, a hallmark of progressive renal disease [28,29]. To investigate the effect of panduratin A on renal interstitial fibrosis in PKD, we measured the amount of collagen deposits in renal tissue after treatment with panduratin A. As shown in Figure 7, treatment with panduratin A (25 mg/kg BW) was able to decrease renal collagen deposition compared to that of vehicle-treated cystic rats. Fibrosis protein markers, including E-cadherin, α-SMA, and p-Smad2/3, were not altered by treatment of panduratin A (25 mg/kg BW).

## 3. Discussion

In addition to a known role in regulating cellular energy metabolism, AMPK activation has been shown to have anti-neoplastic effects [30,31]. Moreover, AMPK expression is reduced in the kidneys of ADPKD rats relative to expression levels in wide-type rats [32], which indicates that AMPK inactivation or inhibition may play a critical role in the pathogenesis of ADPKD. Several studies have shown the potential therapeutic application of modulating AMPK activity in ADPKD [19,20,32,33,34,35]. As such, activation of AMPK by metformin, an anti-diabetic drug, retards cystogenesis via inhibition of cellular proliferation, by targeting the mTOR signaling pathway and CFTR-mediated epithelial fluid secretion [20]. In this regard, by activating AMPK, panduratin A may offer a significant advantage in reducing disease processes. The present study investigated the pharmacological effect and underlying mechanisms of panduratin A, a natural activator of AMPK, on renal cyst enlargement using MDCK cell–derived cyst and PKD rats as in vitro and in vivo models, respectively.

The data presented here demonstrate an inhibitory effect of panduratin A on cystogenesis in MDCK cell–derived cysts. This effect is correlated with AMPK activation as evidence shows that panduratin A stimulates phosphorylation of AMPK. Unfortunately, the development of ADPKD cell–derived cysts was not successful. Therefore, we could not determine the effect of panduratin A on cystogenesis using an ADPKD cell model. The observed inhibitory effect of panduratin A on cyst development could result from decreased cell proliferation or decreased cell viability [36,37]. Our results reveal that panduratin A decreased cell proliferation by attenuating the activity of p70/S6K, which is a downstream target of mTOR signaling pathways in both MDCK and ADPKD cells. Moreover, panduratin A did not reduce the viability of these cells. These data indicate that the inhibitory effect of panduratin A on cyst formation is mediated by abrogating mTOR signaling pathway activity without affecting cell survival. In agreement with the previous study, activation of AMPK slows renal cystogenesis via inhibition of p70/S6K phosphorylation [18]. In addition, our data showed that panduratin A also significantly reduced cyst growth. This effect was mediated by the inhibition of CFTR-mediated chloride secretion but not by altering the transport proteins expressed in the basolateral membrane, including Na^+^–K^+^ ATPase, NKCC1, and K^+^ channels. This is in agreement with previous studies, which found that activation of AMPK by metformin inhibits chloride secretion [20,33,34]. Interestingly, our results demonstcrate that the inhibition of chloride secretion following 24 h treatment did not correlate with a reduction in CFTR protein expression. Interestingly, exposure of the cells to panduratin A for 48 h significantly reduced chloride secretion and CFTR protein expression. These results indicate that the effects of panduratin A on CFTR protein expression are time-dependent. Regulation of CFTR function can be altered by either protein expression or channel gating as well [38,39,40]. Thus, the inhibitory effect of panduratin A on CFTR-mediated Cl^-^ secretion at 24 h might be mediated by reduced CFTR gating, resulting in reduced chloride transport via AMPK activation. This contention is supported by the fact that AMPK decreases CFTR opening by inhibition site of PKA phosphorylation at CFTR [40]. Taken together, the in vitro results illustrate that panduratin A inhibits cell proliferation (inhibition of p70/S6K mTOR pathway) and CFTR function. Therefore, the inhibitory effect of panduratin A on cell proliferation and CFTR-mediated fluid secretion might retard renal cyst progression in PKD.

The potential therapeutic application of panduratin A was also shown in an in vivo model of PKD using (Cy/+) Han:SPRD rats [41,42]. Treatment for 5 weeks with panduratin A at 25 mg/kg BW, but not at 5 mg/kg BW, stimulated phosphorylation of AMPK in renal tissue. Panduratin A reduced cystic progression in (Cy/+) Han:SPRD rats, as shown by decreased cystic area and kidney per bodyweight. The beneficial effects of panduratin A were related to increases in AMPK phosphorylation. Panduratin A showed a tendency to decrease BUN and serum Cr, indicating improved renal function. However, the limitation of this study was that the data were obtained from a small sample size. Increasing the sample size might provide clearer results. As observed in human ADPKD cells, panduratin A inhibits cell proliferation via decreased mTOR/S6K signaling. These results might account for the inhibition of cyst progression. Furthermore, our evidence suggests that panduratin A suppressed phosphorylation of ERK1/2, but not mTOR/S6K, in Han:SPRD rat kidneys. These data are in agreement with a previous study, which found that 2-deoxyglucose (2DG), an analog of glucose that blocks glycolysis, reduced cyst progression in (Cy/+) Han:SPRD rats through the increased phosphorylation of AMPK-α and restoration of ERK signaling in the absence of a reduction in mTOR/S6K [32]. Moreover, metformin was reported to suppress cystogenesis via inhibition of mTOR/S6K signaling in mice models [20]. Therefore, AMPK-driven effects on mTOR/S6K may be dependent on the experimental models. In addition to cyst progression, one of the major pathological changes in polycystic kidney tissues is interstitial fibrosis, which is characterized by excessive collagen production [43]. The present study revealed a beneficial effect of panduratin A on renal fibrosis, as supported by our finding that panduratin A decreased collagen deposition in renal tissue. Interestingly, we could not detect this hallmark change in the expression of proteins involved in renal fibrosis, such as E-cadherin, α-SMA, and Smad2/3. However, our study only measured changes in the early stages of ADPKD, which may explain the lack of effect on the expression of these proteins in (Cy/+) Han:SPRD rats.

Additionally, the efficacy of panduratin A was confirmed in PCK rats, which is a model that resembles human autosomal dominant polycystic kidney disease with slow progression [27]. Panduratin A attenuated cyst enlargement in PCK rats, as shown by the decreased cystic index and the trend of reduced kidney weight. Since the PCK rat model is a slow-progression PKD rat model [41], the beneficial effect of panduratin A was not markedly detected as found in (Cy/+) Han:SPRD rats. In addition, marked increases in BUN and serum Cr levels were not found in PCK rats at 12 weeks of age [44]. Therefore, the beneficial effects of panduratin A on the improvement of renal function were uncertain in this experimental model. To prove whether panduratin A improves renal function in PCK rats, chronic treatment protocols might be required. Although panduratin A treatment (25 mg/kg BW) via i.p. injection shows an efficacy in (Cy/+) Han:SPRD rats, it also produced severe unwanted effects, including internal bleeding. The explanation for this finding is unclear. We suspect that an acute increased plasma concentration of panduratin A from the i.p.-injection route might be the factor responsible. Interestingly, the bleeding was not detected in PCK rats following treatment with 25 mg/kg BW via oral administration for 8 weeks, meaning that the route of administration may be a key factor. Moreover, several studies, and our unpublished data, revealed that treatment with panduratin A via oral administration does not produce severe toxicity [22,45]. Pharmacokinetic data revealed that panduratin A is well absorbed into the circulation following oral administration [46]; therefore, the bioavailability of panduratin A in circulation might not be the major cause of the toxicity following i.p. injection. However, the explanation of why toxicity was induced by the i.p.-injection route is uncertain. This evidence implies that the route of administration might be a major factor in the toxicity of panduratin A. In addition, as the half-life (t_1/2_) of panduratin A is approximately 8 h [46], more frequent oral administration (every 8 h) might produce more beneficial effects in the treatment of PKD than once-daily administration.

In conclusion, panduratin A retarded renal cyst progression via inhibition of cell proliferation. Furthermore, panduratin A reduced renal epithelial secretion of chloride and subsequently decreased cyst expansion via the inhibition of the CFTR transport function. In addition, panduratin A reduced fibrotic markers in ADPKD renal tissue. These findings provide evidential support regarding the potential of panduratin A as a promising candidate for ADPKD treatment.

## 4. Materials and Methods

### 4.1. Preparation of Panduratin A

Dried rhizomes of *Boesenbergia rotunda* (1 kg) were finely ground and extracted with 95% ethanol (7 L) for 7 days at room temperature. Panduratin A was isolated by our previously described method [47]. The purity of panduratin A (>98%) was determined by high-performance liquid chromatography (HPLC) analysis.

### 4.2. Chemicals

The cell culture medium was purchased from Gibco (Grand Island, NY, USA); Dulbecco’s Modified Eagle’s Medium (DMEM)-high glucose (Cat. No. 12800-017) and F-12 Nutrient Mixture (Ham) (Cat. No. 21700-075). Amiloride, forskolin (FSK), and 3-(4,5-dimethyl-2- thiazoyl)-2,5-diphenyl-2H-tetrazolium bromide (MTT) were purchased from Sigma-Aldrich (St. Louis, MO, USA) and 0.25% trypsin–EDTA and penicillin–streptomycin were purchased from Gibco (Grand Island, NY, USA); vasopressin (AVP) (Minirin^®^ inj) was purchased from Ferring GmbH (Kiel, Germany); collagen type I was obtained from Advanced BioMatrix PureCol^®^ (Carlsbad, CA, USA). The bromodeoxyuridine (BrdU) cell proliferation assay kit (Cat. No. QIA58) and phosphatase inhibitor cocktail set II (Cat. No. 524625) were purchased from Calbiochem (Visalia, CA, USA). Protease inhibitor (PI) cocktail (Cat. No. 5892791001) was purchased from Roche (Mannheim, Germany). Primary antibodies for p-S6K (Cat. No. 9204), S6K (Cat. No. 9202), p-ERK1/2 (Cat. No. 9102), ERK1/2 (Cat. No. 9101), p-AMPK (Cat. No. 2531), AMPK (Cat. No. 5832), CFTR (Cat. No. 2269), p-Smad2(Cat. No. 18338), Smad2 (Cat. No. 5339), p-Smad3 (Cat. No. 9520), Smad3 (Cat. No. 9523), E-Cadherin (Cat. No. 3195), GAPDH (Cat. No. 2118), and β-actin (Cat. No. 4970) antibodies were obtained from Cell Signaling (Denvers, MA, USA). Na^+^–K^+^ ATPase (Cat. No. 05-369) antibody was obtained from Merck Millipore (Burlington, MA, USA). α-SMA (Cat. No. ab5694) antibody was obtained from Abcam (Cambridge, UK).

### 4.3. Cell Lines

WT9-12 cells, an immortalized epithelial cell line derived from ADPKD human renal cysts, were obtained from the American Type Culture Collection (ATCC^®^ CRL-2833™; Manassas, VA, USA). Type I Madin–Darby canine kidney (MDCK) cells were kindly provided by Professor David N. Sheppard (University of Walk, Bristol, UK). Both cell lines were maintained at 37 °C in a humidified incubator of 5% CO_2_–95% O_2_ in a 1:1 mixture of Dulbecco’s Modified Eagle’s Medium (DMEM) and Ham’s F-12 nutrient medium supplemented with 10% heat-inactivated fetal bovine serum (FBS), 100 U/mL penicillin, 100 μg/mL streptomycin, and insulin-transferrin-selenium. The culture medium was changed every 2–3 days until the cells reached confluence.

### 4.4. Animals

Han:Sprague–Dawley (Han:SPRD) rats were maintained at the SPF-level laboratory of the animal center at the University of California, Santa Barbara. The study was conducted in heterozygous (Cy/+) and normal littermate control (+/+) Han:SPRD rats. Only male rats were used in this study because the progression of renal cystic disease is very mild in female animals. The rats had free access to water and standard rat chow (PicoLab Rodent Diet 20, #5053). The experimental protocol was approved by the Institutional Animal Care and Use Committee at the University of California, Santa Barbara.

Rats were weaned at 3 weeks of age and separated by sex. A total of 14 rats were divided into 4 groups: (1) control rats (*n* = 3); (2) vehicle-treated (Cy/+) rats (*n* = 4); (3) 5 mg/kg BW of panduratin A–treated (Cy/+) rats (*n* = 4); and 25 mg/kg BW of panduratin A–treated (Cy/+) rats (*n* = 3). Experimental rats were treated daily with either vehicle or panduratin A via intraperitoneal (i.p.) injection, beginning at postnatal week 3 until week 8. At the end of the treatment, all rats were anesthetized by i.p. injection of a ketamine–xylazine combination. All kidneys were removed and weighed, and blood was collected for BUN and serum Cr measurements. For each animal, one kidney was fixed in 10% neutral buffered formalin for 24 h, dehydrated in increasing alcohol, cleared with toluene, paraffinized, and then cut into 5 µm sections for histological examination. The remaining kidney was snap-frozen in liquid nitrogen for further examination of target protein levels by Western blotting.

PCK rats, a spontaneous mutation in a colony of CRJ:CD/SD rats, were obtained from Charles River Japan (Tokyo, Japan). PCK rats, the autosomal recessive polycystic kidney disease (ARPKD) model, show phenotypic PKD [48]. Male rats were employed in this study. Sprague–Dawley (SD) male rats, obtained from Nomura Siam International (Bangkok, Thailand), were used as a control strain. Animals had free access to water and standard diets. Animal experiments were approved by the Faculty of Science, Mahidol University-Institute Animal Care and Use Committee (SCMU-IACUC Protocol no. 63-001-509). Rats were treated once daily by oral gavage with 25 mg/kg BW of panduratin A or vehicle beginning at 4 weeks of age, for 8 weeks. Rats were divided in to 3 groups: (1) control SD rats group (*n* = 4); (2) PCK rats treated with vehicle (*n* = 6); and (3) PCK rats treated with 25 mg/kg BW of panduratin A (*n* = 6). Blood and kidney tissues were harvested at the end of experiment under i.p. of xylazine (15 mg/kg BW) and zoletil (40 mg/kg BW). The samples were assessed for kidney-to-body weight ratios, renal cyst area, BUN, and serum Cr levels.

### 4.5. MDCK Cell-Derived Cyst Formation and Growth

Cyst generation was performed as previously described [16]. Approximately 600 type I MDCK cells were suspended in 0.4 mL of ice-cold 10X Minimum Essential Medium (MEM) (Thermo Fisher Scientific (Thailand), Bangkok, Thailand) containing 3.1 mg/mL collagen, 10 mM HEPES, 27 mM NaHCO_3_, 100 U/mL penicillin, and 100 μg/mL streptomycin (pH 7.4 with NaOH) and added to individual wells of a 24-well plate. Cell-embedded collagen gels were maintained at 37 °C in a 5% CO_2_-humidified atmosphere for about 90 min to allow gelation of the collagen. After gelation, 1.5 mL of MDCK cell medium containing 10 μM forskolin was added to each well to promote cyst formation. To quantify cyst formation, the number of cysts with diameters ≥ 50 μm and non-cyst cell colonies were counted on day 6 using a phase-contrast microscope (×10 magnification). To test MDCK cyst growth, at least 20 cysts with diameters ≥ 50 μm were imaged 10× magnification on days 6, 8, 10, and 12 using a phase-contrast microscope. Cyst diameters (µm) were measured using Image J software.

### 4.6. Cell Viability Assay

Cells in a 96-well plate were exposed to an MTT solution (0.5 mg/mL) for 4 h in a humidified atmosphere of 5% CO_2_, 95% O_2_ at 37 °C. The medium was then removed and 100 μL DMSO was added to the well plates. The absorbance at 570 nm was detected using an Envision microplate reader (Perkin Elmer, Waltham, MA, USA). Cell viability was reported as a percentage of the vehicle-treated group.

### 4.7. Cell Proliferation Assay

Cell proliferation assays were performed using a BrdU cell proliferation assay kit. MDCK and WT9-12 cells were seeded into 96-well plates at a density of 6 × 103 cells per well and grown for 24 h in DMEM/Ham’s F-12 media supplemented with 1% FBS and ITS supplement. After treatment, BrdU reagent solution was added at 6 h before the end of the incubation time. Cells were then fixed using fixative/denaturing solutions and stained with anti-BrdU antibodies and goat anti-mouse IgG horseradish peroxidase (Cat. No. QIA58). After the addition of a substrate solution in the dark, a stop solution was added, and absorbance was measured at 450 nm and 490 nm using a microplate reader. Cell proliferation was reported as the percent of cell proliferation in the experimental group compared to the vehicle-treated group.

### 4.8. Real-Time PCR

Total RNA was extracted from MDCK cells using TRIzol according to the manufacturer’s instructions. RNA content was measured using a Nanodrop spectrophotometer. Next, RNA was converted into cDNA by using the iScript cDNA synthesis kit (Bio-Rad). The cDNA was then used as a template for real-time PCR with gene-specific primers. Target gene–specific primers (forward and reverse) were designed based on their sequences in the National Center for Biotechnology Information (NCBI) database.

The following primers were used: canine K^+^ channel (KCa3.1) (forward, 5-GCAAGA- GGCCTGGATGTTCT-3 and reverse, 5-AACACACAGGTGTCTCGACC-3); canine NKCC1 (Na^+^-K^+^-2Cl^−^) (forward, 5-AGGATGGCAAGACTCCAACTC-3 and reverse, 5-AAAGTAG CCATCGCTCTCCG-3); and canine GAPDH (forward, 5-CCATGTTTGTGATGGGCGTG-3 and reverse, 5-CATGGACGGTGGTCATGAGG-3). Quantitative PCR of the target mRNA expression level was performed using the SYBR Green Master Mix. The complete reactions were subjected to the following program of the thermocycling conditions: 95 °C for 5 min, 95 °C for 1 min, 30 cycles of 60 °C for 1 min and 75 °C for 60 s, and 10-min final heating at 72 °C. The melting curve was run after the PCR cycles to check the specificity of each primer. Data were analyzed by normalizing the expression of mRNA levels of target genes to the expression level of GAPDH as a housekeeping gene.

### 4.9. Western Blot Analysis

MDCK or WT9-12 cells grown in 6-well plates or Petri dishes (60 mm × 15 mm) were lysed with ice-cold lysis buffer containing 1% Triton X-100, 50 mM Tris-HCl pH 7.4, 150 mM NaCl, 1 mM EDTA, 1 mM NaF, 1 mM Na_3_VO_4_, 1 mM PMSF (phenylmethylsulfonyl fluoride), a protease inhibitor (PI) cocktail, and a phosphatase inhibitor cocktail set II for 20 min at 4 °C. For kidney lysates, kidney cells were lysed, and proteins were extracted using a cold lysis buffer containing 0.3 M sucrose, 25 mM imidazole, 1 mM EDTA, 1 mM PMSF, PI cocktail, and phosphatase inhibitor. The lysis buffer containing the cell lysates was subsequently centrifuged at 10,000× *g* for 20 min at 4 °C. The supernatant proteins were collected and loaded in an 8–10% Tris–Glycine SDS–PAGE gel and then transferred to a nitrocellulose membrane. Membranes were blocked with 5% non-fat dry milk in Tris-buffered Saline–Tween 20 (TBST) for 90 min at room temperature before incubation overnight at 4 °C with antibodies against proteins of interest. After washing, membranes were incubated with secondary HRP-conjugated anti-rabbit or anti-mouse IgG antibodies for 1 h at room temperature. The immunoblot was detected by using a chemiluminescence detection method. Band intensities were quantified using Image J analysis software.

### 4.10. Ussing Chamber Experiments

Isc was used to monitor net ion flux across MDCK cell monolayers, as previously described [9]. MDCK cell monolayers with high resistances (transepithelial resistance >1000 Ω·cm^2^) were mounted on an Ussing chamber system (Physiological Instruments, San Diego, CA, USA). Both hemichambers were filled with Krebs’ buffer solutions containing (in mM) 117 NaCl, 25 NaHCO_3_, 4.7 KCl, 1.2 MgSO_4_, 2.5 CaCl_2_, 1.2 KH_2_PO_4_, and 11 D-glucose at 37 °C and bubbled with a gas mixture of 95% O_2_–5% CO_2_ to maintain a pH of 7.4. To stimulate CFTR-mediated chloride secretion, 20 nM AVP was added to the basolateral side after the addition of 100 µM amiloride at the apical side to inhibit sodium transport via epithelial sodium channels (ENaC).

Measurement of Na^+^–K^+^ ATPase transport activity; The transport activity of Na^+^–K^+^ ATPase was measured according to the previously described method [9]. Apical and basolateral compartments were bathed in Krebs’ buffer solutions maintained at 37 °C and bubbled with a gas mixture of 95% O_2_–5% CO_2_. The apical membrane of the monolayer was permeabilized by 250 µg/mL amphotericin B to allow the free transport of ions into the cell from the apical side. Ouabain (1 mM), an inhibitor of Na^+^–K^+^ ATPase, was added to the basolateral side of monolayers after the current (Isc) became stable. The change in Isc after the addition of ouabain reflected the activity of Na^+^–K^+^ ATPase.

### 4.11. Histological Staining

Kidneys were fixed in 10% neutral buffered formalin, embedded in paraffin, and sectioned. Kidneys were sectioned at 5 µm and stained with hematoxylin and eosin (H&E). All kidney slides were captured by an inverted contrast phase light at 10× magnifications. For cystic measurement, images of H&E sections were overlaid with a grid in Adobe Photoshop and intersection points on cysts vs. normal tissue were counted. For fibrosis measurement, sections were deparaffinized and stained with 0.1% Sirius Red and 0.1% Fast Green. The percentage of collagen was quantified as the percentage of red colored area. The measurement was performed using Image J software by setting thresholds specific for the color red. Representative images of Sirius Red staining were captured at 400× magnification.

### 4.12. Statistics

All results were analyzed with GraphPad Prism software and represented as mean ± SD. The statistical significance of the data between control and treatment groups was determined using a one-way ANOVA followed by a Tukey’s multiple comparison tests. Two-tailed Student’s *t* tests were used to determine significance between two sets of data. A level of *p* < 0.05 was considered statistically significant.

## Data Availability

Not applicable.

## Supplementary Materials

The following are available online at https://www.mdpi.com/article/10.3390/ijms23084328/s1.
