# Supplementary material for: Pharmacological Effects of Panduratin A on Renal Cyst Development in In Vitro and In Vivo Models of Polycystic Kidney Disease
Source: Int J Mol Sci. 2022 Apr 14;23(8):4328. doi: 10.3390/ijms23084328 (PMC9024631; doi:10.3390/ijms23084328)
Supplement: Supplementary file 1 [file ijms-23-04328-s001.zip › ijms-1643705-supplementary.pdf]

Supplement data

Table S1 Body weight of (Cy/+) Han:SPRD rats after 5 weeks of treatment

Group	Body weight (g) (week 0)	Body weight (g) (week 5)
(+/+) Vehicle (n = 3)	49.00 ± 7.81	286.00 ± 27.84
(Cy/+) Vehicle (n = 4)	52.00 ± 6.88	295.00 ± 18.00
(Cy/+) Pan A 5 (n = 4)	46.25 ± 6.50	264.50 ± 10.08
(Cy/+) Pan A 25 (n = 3)	54.67 ± 3.06	218.33 ± 12.86**

** $P < 0.01$ vs vehicle control of (+/+) and (Cy/+)

Table S2 Body weight of SPD and PCK rats after 8 weeks of treatment

Group	Body weight (g) (week 0)	Body weight (g) (week 8)
SPD (Vehicle) (n = 4)	83.50 ± 7.05	495.00 ± 13.83
PCK (Vehicle) (n = 5)	82.00 ± 12.47	438.40 ± 14.93***
PCK (Pan A 25) (n = 6)	82.50 ± 12.76	444.67 ± 14.61***

*** $P < 0.001$ vs vehicle control of SPD
